# Phase I clinical trial of a novel autologous modified-DC vaccine in patients with resected NSCLC

**DOI:** 10.1186/s12885-017-3859-3

**Published:** 2017-12-21

**Authors:** Chunlei Ge, Ruilei Li, Haifeng Song, Tao Geng, Jinyan Yang, Qinghua Tan, Linfeng Song, Ying Wang, Yuanbo Xue, Zhen Li, Suwei Dong, Zhiwei Zhang, Na Zhang, Jiyin Guo, Lin Hua, Siyi Chen, Xin Song

**Affiliations:** 1 Cancer Biotherapy Center, The Third Affiliated Hospital of Kunming Medical University (Tumor Hospital of Yunnan Province), Kunming, Yunnan 650118 China; 2 Center for Cell and Gene Therapy, Baylor College of Medicine, Houston, TX USA; 3 Department of Immunology, Baylor College of Medicine, Houston, TX USA; 4 Department of Molecular and Human Genetics, Baylor College of Medicine, Houston, TX USA

**Keywords:** Modified-DCvaccine, Non-small cell lung cancer (NSCLC), Phase I clinical trial

## Abstract

**Background:**

The primary aim of this study was to evaluate the safety of a novel dendritic cell (DC) vaccine pulsed with survivin and MUC1, silenced with suppressor of cytokine signaling 1 (SOCS1), and immune stimulated with flagellin for patients with stage I to IIIA non-small cell lung cancer (NSCLC) in a phase I open-label, uncontrolled, and dose-escalation trial. Moreover, we evaluate the potential efficacy of this modified DC vaccine as secondary aim.

**Methods:**

The patients were treated with the vaccine at 1 × 10^6^, 1 × 10^7^and the maximum dose 8 × 10^7^ at day 7, 14, and 21 after characterization of the vaccine phenotype by flow cytometry. The safety of the vaccine was assessed by adverse events, and the efficacy by the levels of several specific tumor markers and the patient quality of life.

**Results:**

The vaccine was well tolerated without dose-limiting toxicity even at higher doses. The most common adverse event reported was just grade 1 flu-like symptoms without unanticipated or serious adverse event. A significant decrease in CD3 + CD4 + CD25 + Foxp3+ T regulatory (Treg) cell number and increase in TNF-α and IL-6 were observed in two patients. Two patients showed 15% and 64% decrease in carcino-embryonic antigen and CYFRA21, respectively. The vaccination with the maximum dose significantly improved the patients’quality of life when administered at the highest dose. More importantly, in the long-term follow-up until February 17, 2017, 1 patient had no recurrence, 1 patients had a progressive disease (PD), and 1 patient was died in the low dose group. In the middle dose group, all 3 patients had no recurrence. In the high dose group, 1 patient was died, 1 patient had a PD, and the other 7 patients had no recurrence.

**Conclusions:**

We provide preliminary data on the safety and efficacy profile of a novel vaccine against non-small cell lung cancer, which was reasonably well tolerated, induced modest antitumor activity without dose-limiting toxicity, and improved patients’ quality of life. Further more, the vaccine maybe a very efficacious treatment for patients with resected NSCLC to prevent recurrence. Our findings on the safety and efficacy of the vaccine in this phase I trial warrant future phase II/III clinical trial.

## Background

Lung cancer is the leading cause of cancer mortality in both men and women, accounting for 1.2 million deaths and 1.6 million total cases in 2008 [1]. The incidence of new cases and deaths from lung cancer are increasing worldwide [2]. Non–small cell lung cancer (NSCLC) accounts for 85% of lung cancer cases with a 16% 5-year survival rate for all stages [3]. Surgery following platinum-based chemotherapy and radiation are still the primary treatment for resectable stage I to IIIA NSCLC, with the five-year survival being 19%–50% [4]. Second-line therapy, such as Pemetrexed and Docetaxel result in slightly better survival rates, and targeted agents such as gefitinib, erlotinib, crizotinib, and bevacizumab result in prolonged overall survival or progression-free survival. However, only a small group of patients are sensitive to these targeted agents [5–10], calling for the development of new strategies against NSCLC.

Immunotherapy is an inspiring systemic strategy for provoking the immune system to attack patient tumor cells [11]. Dendritic cells (DCs) as “gatekeepers of the immune system” are the most potent antigen-presenting cells, and numerous clinical trials have shown that DC-based cancer vaccines can induce successful therapeutic and protective immune response. Notably, Provenge, a prostate cancer vaccine, exhibited promising outcomes using autologous DC pulsed with fusion antigen protein consisting of prostatic acid phosphatase (PAP) and GM--CSF, the first therapeutic cancer vaccine to be approved by the U.S. Food and Drug Administration in 2010, showed to prolong median OS by 4.1 months for metastatic castration resistant prostate cancer [12]. Since then, several DC vaccine clinical trials in patients with malignant glioma [13], metastatic melanoma [14] advanced hepatocellular carcinoma [15] and esophageal cancer [16, 17] have been reported. Although some of these trials did not reach the end point of primary study, others have reported positive results. Among factors that influence DC antigen presentation, such as the number, maturity state, and peptides used to pulse DC, a right peptide is the most important for a successful DC vaccine.

A large number of studies have shown that the tumor antigens survivin and MUC1 are highly expressed in variety of tumors, especially lung cancer. Survivin as a member of the inhibitor of apoptosis protein (IAP) family plays a pivotal role in inhibiting apoptosis and regulating cell division. The over-expression of surviving is correlated with unfavorable clinical outcome in many tumor types, including NSCLC [18–23]. Survivin is expressed in at least 80% of tumor patients with NSCLC, and the suppression of surviving expression abrogates survivin-mediated apoptosis, which results in increased in tumor-cell death and eventually sensitivity to anticancer therapy [24]. MUC1, a heavily glycosylated large glycoprotein, is frequently over-expressed on the cell surface of glandular epithelial cells in a variety of tumor types, including NSCLC [25, 26]. MUC1 is involved in tumorigenesis and invasiveness by modulating cell adhesion [27]. For examples, several studies have demonstrated that MUC1 expression is associated with a poor prognosis in NSCLC [28], and a number of clinical trials using MUC1 pulsed DC demonstrated positive immune response in patients with pancreatic and biliary tumors [29, 30]. However, single-antigen-loaded DCs are not sufficient to elicit stronger enough cytotoxic T-lymphocyte (CTL) response due to heterogeneity of cancer cells, whereas MUC4 and survivin-loaded DCs have been shown to successfully induce stronger CTL responses against pancreatic cancer in vitro [31]. Therefore, the combination of survivin and MUC1 may offer a new strategy for development of a DC cancer vaccine.

Interestingly, down-regulation of suppressor of cytokine signaling 1 (SOCS1), which is an attenuator of cytokine signals, promotes memory T cell responses in dendritic cells [32]. A SOCS1 suppressor antagonist enhances antigen-presenting capacity and tumor cell antigen-specific cytotoxic T lymphocyte responses [33, 34]. Therefore, SOCS1 plays an essential role in negative regulation of DC antigen presentation and inhibition of DC differentiation and induces immune tolerance [35]. In fact, inhibition of SOCS1 breaks self-immune tolerance and induces effective antitumor responses [36, 37] and anti-HIV effects [38]. Furthermore, SOCS1 inhibits Toll-like receptor (TLR) signaling [39]. While the SOCS1 function in carcinogenesis among different cancer cells is still controversial, it has been suggested that modulation of SOCS1 expression in tumor cells for antitumor therapy is highly context-dependent [40]. Further studies are warranted to understand the role of SOCS1 in suppressing NSCLC.

Inhibition of SOCS1 alone is insufficient to fully activate DCs [41]. TLR signaling is important for triggering and modulating adaptive immune response through activation of DCs [42, 43]. Flagellin, a specific ligand for TLR5, plays an important role in activating immune response via triggering TLR signaling [44, 45]. A bacterial filament protein, flagellin incombination with siRNA-SOCS1 modified-DC vaccine was found to be more potent and persistent than a commercial TLR agonist in both murine and human DCs and displays a superior ability to activate HCV antigen-specific cellular and humoral immune responses [46, 47].

However, there is no DC vaccine pulsed with survivin and MUC1, silenced with SOCS1, and immune stimulated with flagellin, especially in the context of vaccine against NSCLC vaccine. Here, we evaluated the safety and efficacy of a novel modified-DC vaccine in patients with stage I to IIIA NSCLC.

## Methods

### Eligibility criteria

Patients with histologically confirmed stage I to IIIA NSCLC were eligible for the phase I clinical trial. The inclusion criteria included: age between 18 and 65 years; an Eastern Cooperative Oncology Group performance status of 0 or 1; life expectancy more than 6 months; adequate bone marrow function (e.g., total white blood cells ≥2000/mm^3^, hemoglobin ≥9 g/dL, granulocyte count >1000/mm^3^, and platelet count ≥100,000/mm^3^); adequate liver function (total serum bilirubin <1.5 mg/dl, aspartate aminotransferase, and alanine aminotransferase ≤5 times upper limit of normal); adequate renal function (serum creatinine <2.0 mg/dl and/or creatinine clearance ≥60 mL/min); major surgery, chemotherapy, radiotherapy, or immunotherapy terminated at least 6 weeks and recovery from the toxic effects of these treatments; positive for histochemical staining of both survivin and MUC1 in tumor regions. Patients were excluded if they had a clinically significant cardiac abnormalities, severe cardiovascular, decompensated heart insufficiency, ventricular rhythm disorders, coagulation disorders, active inflammatory disease, positive for hepatitis B/C or HIV, history of an autoimmune disease (e.g., systemic lupus, rheumatoid arthritis, and others), severe psychiatric disease, known immunosuppressive disease or use of immunosuppressive drugs (steroids), or history of other neoplasms, and pregnancy or lactation. Histologic type and Tumor Node Metastasis (TNM) classification were classified, according to the criteria of the American Joint Commission on Cancer (AJCC) [48].

Written informed consent was obtained from all the patients. This study was approved by the ethics committee of the Third Affiliated Hospital of Kunming Medical University and was carried out in accordance with criteria of Good Clinical Practice (GCP). The ethical approval reference number is KY2009-iAPA.

### Generation of modified-DC vaccine

DCs were generated from peripheral blood mononuclear cells (PBMCs) from each patient using the Cobe Spectra Apheresis System (GambroBCT, USA). PBMCs were cultured for six days in serum-free, GMP (Good Manufacturing Practice) certified medium supplemented with granulocyte-macrophage colony-stimulating factor (GM-CSF) and interleukin-4 (IL-4) to obtain immature dendritic cells (iDC). The PBMCs were isolated by leukapheresis,and then re-suspended in serum-free media at 37 °C in a humidified 5% CO_2_/95% air incubator. After incubation for half an hour, non-adherent cells were removed, and the adherent cells were cultured in media supplemented with 30 ng/mL recombinant human interleukin 4 (IL-4, Proteck, R&D systems, USA), 100 ng/mL recombinant human granulocyte macrophage-colony stimulating factor (GM-CSF; R&D systems, USA), and 2% human serum albumin and 2 mmol/L glutamine for 6 days. Fresh media supplemented with the cytokine were added every other day. Then iAPA cytokine (10,000 vp/cell) that includes SOCS1-specific small interfering RNA and peptides for Flagellin, survivin, and MUC1 were added to the culture at day 6. The cultured DCs were harvested by vigorous washing with sterile 0.9% NaCl solution at day 7. All the DCs were tested for bacterial, fungal, mycoplasma, and endotoxin and viability prior to vaccination. The matured DCs were confirmed using flow cytometry analysis before vaccination were harvested, washed and re-suspended in 100 mL of sterile 0.9% NaCl solution containing 1% serum albumin (Baxter, Austria).

### Vaccination of the modified-DC vaccine

**Fig. 1:**
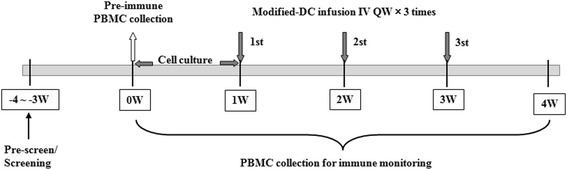
Modified-DC vaccination schedule. W, week; IV, intravenous; PBMC, peripheral blood mononuclear cell

The protocol was an open-label, uncontrolled, and dose-escalation phase I trial. This phase I trial was not retrospectively registered in ClinicalTrials.gov. Patients were intravenously injected with 1 × 10^6^, 1 × 10^7^ and the maximum dose DC vaccine suspended in 100 mL of sterile 0.9% NaCl solution containing 1% serum albumin at day 7, 14 and 21, respectively (Fig. 1). Dose escalation proceeded using a 3 + 3 cohort design [49]. First, according to eligibility criteria, 3 patients were enrolled and divided into low dose groups, who were intravenously injected with 1 × 10^6^ DC vaccine. Adverse events (AEs) were observed at 0, 0.5, 1, 1.5, 2, 4, 6, 12, 24, 48, and 72 h after each injection using Common Terminology Criteria for Adverse Events version 4 (CTCAE 4). If there were no unanticipated or serious adverse events occurred in the 28-day period, 3 another patients were enrolled into the middle dose group for 1 × 10^7^ DC vaccine. AEs were also observed, and if there were no unanticipated or serious adverse events, then enrolled 9 patients into the high dose group for the maximum dose DC vaccine. If there were unanticipated or serious adverse events occurred in any stage, the clinical trial would be ended.

### Immunohistochemical staining

Paraffin-embedded NSCLC lung samples were cut to 4 μm. All sections were baked at 70 °C for 1 h, hydrated with xylene and alcohol as routine, and microwaved in a citrate buffer (pH 6.0) for 5 min for antigen retrieval. Endogenous peroxidase activity was blocked with 0.3% hydrogen peroxide for 30 min. Then the sections were incubated with mouse monoclonal anti-survivin (1:10 R&D Systems, Minneapolis, MN, USA) and anti-MUC1 (1:200, Abcam, Cambridge, UK) in phosphate-buffered saline(PBS; pH 7.4) overnight, followed by washing three times with PBS. The slides were incubated with an ABC kit (Vector, Burlingame, CA, USA), according to the manufacturer’s instructions, color developed with 3–3′-diaminobenzidine (DAB; Dako Corporation, Carpinteria, CA, USA), and counter stained with hematoxylin. Gastric carcinoma section was used as positive control, and the pre-immune serum was used as a negative control.

### Evaluation of survivin and MUC1 expression

Survivin staining was evaluated, according to semi-quantitative method [20]. The staining intensity was graded as follows: 1+ (weak), 2+ (moderate), and 3+ (intense). The grade of positive staining (mean percentage) was assigned as follows: 0 (< 5%), 1 (5–25%), 2 (26–50%), 3 (51–75%), and 4 (>75%). The two grades were then multiplied to derive a score for each sample. MUC1 staining was evaluated, according to semi-quantitative analysis [50] as follows: (negative), 1+ (weak), 2+ (moderate), and 3+ (intense). The positive staining (mean percentage) for MUC1 was assigned as follows: 1 (<10%), 2 (10–50%), and 3 (51–100%). Scoring was independently performed by two pathologists blinded to clinical outcome and reached a consensus for all slides.

### Analysis of DC phenotype by flow cytometry

The purity and phenotype of modified-DC was analysed using FACS Canto II flow cytometer (BD Biosciences, USA). Cells were stained with fluorescein isothiocyanate(FITC)-conjugated CD86, phycoerythrin(PE)-conjugated CD80, CD14, and HLA-DR, allophycocyanin(APC)-conjugated CD83, CD40, CD54, and HLA-ABC monoclonal antibodies (BD Biosciences, USA), and PE-conjugated CCR7 monoclonal antibody (R&D systems, USA). FITC-, APC-, PE- mouse isotype immunoglobulins, and PE mouse anti-human HLA-DR were used as background controls.

### Lymphocyte populations

T cell subsets, including CD3^+^CD4^+^, CD3^+^CD8^+^, CD3^+^Vα24^+,^ CD3^+^CD56^+^, and CD3^+^CD4^+^CD25^+^FoxP3^+^, and NK cell CD16^+^CD56^+^ in PBMCs were analysed using FACS Canto II flow cytometer prior vaccination to vaccination on day 14 (7 days post the first vaccination), day 21(7 days post the second vaccination), and day 28(7 days post the third vaccination). Cells were stained with FITC-labeled CD3, CD4, and CD56, APC- labeled CD3, CD4, andCD25, PE-labeled CD8, CD16 + CD56, FoxP3, and TCR-Vα24, and PerCP-labeled CD45 antibodies (BD Biosciences, USA). The data were analysed by Cell Quest software (FACS Diva, BD Biosciences, USA). FITC-, APC-, PE-, and PerCP- mouse isotype immunoglobulins were used as background controls for nonspecific immunofluorescence.

### Patient assessment

Adverse events (AEs) were observed at 0, 0.5, 1, 1.5, 2, 4, 6, 12, 24, 48, and 72 h after each injection using Common Terminology Criteria for Adverse Events version 4 (CTCAE 4). Clinical responses were evaluated using computed tomography (CT) at one week after the last modified DC vaccine was injected. Immunologic responses and tumor markers were assayed before injection of the modified-DC vaccine at day 7, 14, 21, and 28. Health-related quality of life was evaluated by the EORTC QLQ-C30 Scoring Manual before vaccination and at one week after the last injection. Function of vital organs was monitored by laboratory data and electrocardiography (ECG).

### Cytokine secretion assay

The changes of interleukin-2 (IL-2), IL-4, IL-6, and IL-10, interferon gamma (IFN-γ), and tumor necrosis factor alpha (TNF-α) in the sera of the patients were assessed at day 0, 14, 21, and 28. Sera from the patients were stored at −20 °C before measuring cytokine levels using a human Th1/Th2 cytokine kit II (BD Biosciences, USA) by Cytometric Bead Array (CBA).

### Statistical analysis

Statistical analyses were performed using SPSS16.0 (SPSS Inc., Chicago, IL, USA). Modified-DC phenotypes were expressed as mean ± SD and analysed by the paired-samples *t*-test for normal distribution. Wilcoxon matched-pairs signed-ranks test was used for non-normal distribution. Tumor markers, cytokines, and T cell populations were determined by repeated measurement analysis of variance (ANOVA). Wilcoxon rank sum test was used to analyze Quality-of-Life. A *p*-value <0.05 was considered statistically significant.

## Results

### Patient characteristics

**Fig. 2:**
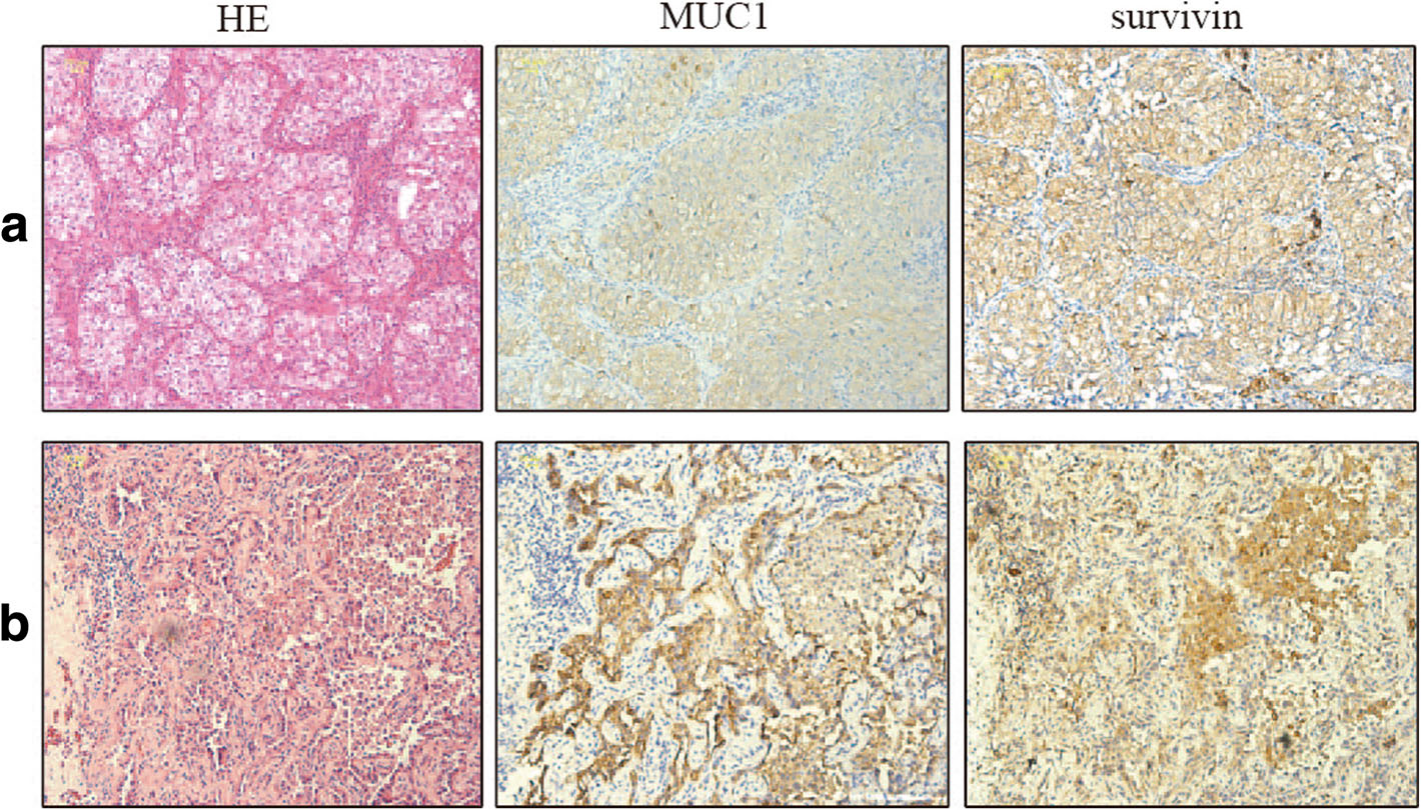
Immunohistochemical expression of survivin and MUC1 in NSCLC [×10]. Survivin and MUC1 staining was carried out in the same NSCLC tumor biopsies. Survivin was expressed mainly in the cytoplasm, and MUC1 was mainly expressed in the membrane. **a** Patient 9 with squamous cell carcinoma. **b** Patient 1 with adenocarcinoma. Scale bar: 20 μm

15 patients with resected stage I to IIIA NSCLC were enrolled in this dose-escalation study from August 2012 to September 2013. Three patients were treated with 1 × 10^6^ of modified-DC vaccines; another three patients were treated with 1 × 10^7^; and nine patients were treated with maximum dose 8 × 10^7^ of modified-DC vaccines. Patient characteristics are shown in Table 1. Median age was 50 years (range from 40 to 61 years), and 15 patients had Eastern Cooperative Oncology Group Performance Status (ECOG PS) grade 0. The histologic subtypes of patients include adenocarcinoma, squamous carcinoma, and adenosquamous carcinoma. Immunohistochemical analysis of survivin and MUC1 expression in the tumor biopsies prior to vaccination were both positive in all patients. Survivin expression was observed mainly in the cytoplasm and a little in the nucleus and cytoplasmic membrane, with staining scores of 1+, 2+ and 3+ in 4 (26.7%), 9 (60%), and 2 (13.3%) specimens, respectively. MUC1 expression was observed mainly in themembrane, with staining scores of 2+ and 3+ in 9 (60%), and 6 (40%) specimens, respectively (Fig. 2).

**Table 1:** Patient Characteristics (n = 15)

Characteristics	No. of Patients	%
Sex		
Female	3	20
Male	12	80
Age, years		
Median	50	
Range	40–61	
ECOG PS		
0	15	100
Histologic subtypes		
Adenocarcinoma	10	66.7
Squamous carcinoma	4	26.7
Adenosquamous carcinoma	1	6.7
Tumor stage (AJCC)		
IA	4	26.7
IB	6	40
IIA	2	13.3
IIB	1	6.7
IIIA	2	13.3
Lymph node metastasis		
Yes	2	13.3
No	13	86.67
History of chemotherapy		
Yes	12	80
No	3	20
History of radiotherapy		
Yes	0	0
No	15	100

*Abbreviations*; *ECOG* Eastern Cooperative Oncology Group, *PS* Performance status

### Safety and toxicity

Before injected into patients, we detect the safety and the viability of the DC vaccines. All Trypan blue viability was ≥70%, and for there is no growth for bacterial and fungal, endotoxin assay is <5 EU/mL, and Mycoplasma (PCR) is negative. Dose-limiting toxicity was not observed by vaccination with the modified-DC vaccines. The most common adverse events (AEs) were grade 1 flu-like symptoms which did not require any intervention, including pyrexia (40%), fatigue (33.33%), C-reactive protein (CRP) increased (46.67%), myalgia (40%), abdominal pain (33.33%), and nausea (20%). Pyrexia and myalgia commonly occurred in the group immunized with the maximum amount of the vaccine in 5 of 9, and 4 of 9 patients, respectively. One of the patients in the maximum amount vaccine immunized group developed the Grade 1 pyrexia and had the highest temperature 38.9 °C within 4 to 10 h, but the temperature decreased to normal levels within 10 to 20 h after the vaccine infusion. No unanticipated or serious adverse events occurred in the 28-day period. The numbers of vaccination-related AEs are summarized in Table 2a-b.

**Table 2:** National Cancer Institute Common Toxicity Criteria for Adverse Events in study population (n = 15)

A									
Adverse Event		Total, n (%)							
		N = 15							
		Any Grade			Grade 1–2		Grade 3–4		
Pyrexia		6 (40%)			6 (40%)		0		
Fatigue		5 (33.33%)			5 (33.33%)		0		
Palpitate		1 (6.67%)			1 (6.67%)		0		
Headache		3 (20%)			3 (20%)		0		
Chest pain		1 (6.67%)			1 (6.67%)		0		
Chest Congestion		1 (6.67%)			1 (6.67%)		0		
Abdominal pain		5 (33.33%)			5 (33.33%)		0		
Abdominal distension		2 (13.33%)			2 (13.33%)		0		
Nausea		3 (20%)			3 (20%)		0		
Hypertension		1 (6.67%)			1 (6.67%)		0		
Nasal congestion		1 (6.67%)			1 (6.67%)		0		
CRP increased		7 (46.67%)			7 (46.67%)		0		
Myalgia		6 (40%)			6 (40%)		0		
Creatinine increased		2 (13.33%)			2 (13.33%)		0		
Chills		1 (6.67%)			1 (6.67%)		0		
B									
Adverse Event	1 × 10^6^, n (%)			1 × 10^7^, n (%)			Maximum numbersof cultured cell, n (%)		
	*N* = 3			N = 3			*N* = 9		
	Any Grade	Grade 1–2	Grade 3–4	Any Grade	Grade 1–2	Grade 3–4	Any Grade	Grade 1–2	Grade 3–4
Pyrexia	0	0	0	1 (33.33%)	1 (33.33%)	0	5 (55.56%)	5 (55.56%)	0
Fatigue	1 (33.33%)	1 (33.33%)	0	1 (33.33%)	1 (33.33%)	0	3 (33.33%)	3 (33.33%)	0
Palpitate	1 (33.33%)	1 (33.33%)	0	0	0	0	0	0	0
Headache	1 (33.33%)	1 (33.33%)	0	1 (33.33%)	1 (33.33%)	0	1 (11.11%)	1 (11.11%)	0
Chest pain	1 (33.33%)	1 (33.33%)	0	0	0	0	0	0	0
Chest Congestion	1 (33.33%)	1 (33.33%)	0	0	0	0	0	0	0
Abdominal pain	3 (100%)	3 (100%)	0	0	0	0	2 (22.22%)	2 (22.22%)	0
Abdominal distension	0	0	0	1 (33.33%)	1 (33.33%)	0	1 (11.11%)	1 (11.11%)	0
Nausea	1 (33.33%)	1(33.33%)	0	0	0	0	2 (22.22%)	2 (22.22%)	0
Hypertension	0	0	0	1(33.33%)	1(33.33%)	0	0	0	0
Nasal congestion	1 (33.33%)	1 (33.33%)	0	0	0	0	0	0	0
CRP increased	2 (66.67%)	2 (66.67%)	0	1 (33.33%)	1 (33.33%)	0	4 (44.44%)	4 (44.44%)	0
Myalgia	1 (33.33%)	1 (33.33%)	0	1 (33.33%)	1 (33.33%)	0	4 (44.44%)	4 (44.44%)	0
Creatinine increased	0	0	0	2 (66.67%)	2 (66.67%)	0	0	0	0
Chills	1 (33.33%)	1 (33.33%)	0	0	0	0	0	0	0

### Modified-DC phenotypes

**Fig. 3:**
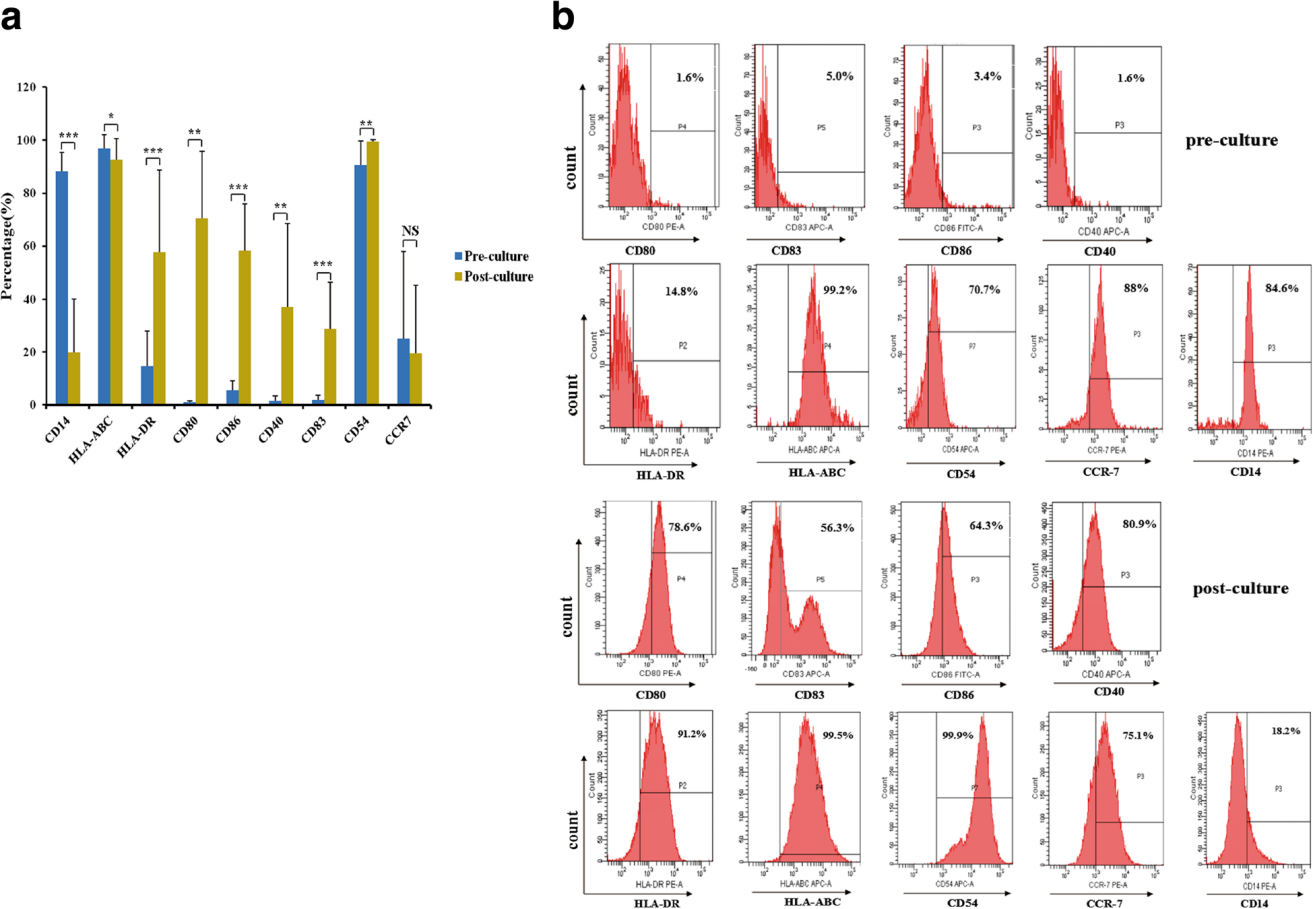
The phenotypes of Modified-DC (HLA-ABC/HLA-DR/CD80/CD86/ CD40/CD83/CD54/CCR7) by flow cytometry analysis. **a** The pooled data (*n* = 15) represents modified-DC phenotypes of pre- and post-culture. Results represent mean ± standard deviation. The cell population with HLA-DR/CD80/CD86/CD40 /CD83/CD54 phenotypein matured modified-DCs were significantly increased compared with pre-culture (n = 15; *, *p* < 0.05; **, *p* < 0.01; ***, *p* < 0.00; NS, no significant); the cell population with HLA-ABC and CCR7 phenotype in matured modified-DCs were decreased compared with pre-culture (n = 15, *p* < 0.05 and *p* > 0.05, respectively). **b** Representative data of different cell populations in matured and pre-cultured DCs

Final modified-DC sup-regulated immunostimulatory molecule expression on the cell surface. The number of cell populations with CD14 expression was down-regulated from 88.3 ± 7.08% to 19.88 ± 20.28%; the cell population with HLA-ABC expression was down-regulated from 97.01 ± 5.05% to 92.5 ± 8.14%; the cell population with HLA-DR expression was up-regulated from 14.67 ± 13.21% to 57.75 ± 30.93%; cell population with co-stimulatory molecule CD80, CD86, and CD40 was dramatically up-regulated, from 0.99 ± 0.58% to 70.46 ± 25.37%, 5.71 ± 3.46% to 58.24 ± 17.66%, and 1.57 ± 1.90% to 37.02 ± 31.59%, respectively; the cell population with the expression of maturation marker, CD83 and CD54, was also up-regulated from 1.91 ± 1.81 to 28.83% ± 17.57 and 90.6 ± 9.09% to 99.51 ± 0.64%, respectively. Meanwhile, the expression of C-chemokine receptor 7 (CCR7) which induces DC maturation and activation signals remained almost unchanged from 25.15% ± 32.81 to 19.55% ± 25.78 (*p* > 0.05). The cell populations associated with modified-DC are shown in Fig. 3.

### Patient immune responses

**Fig. 4:**
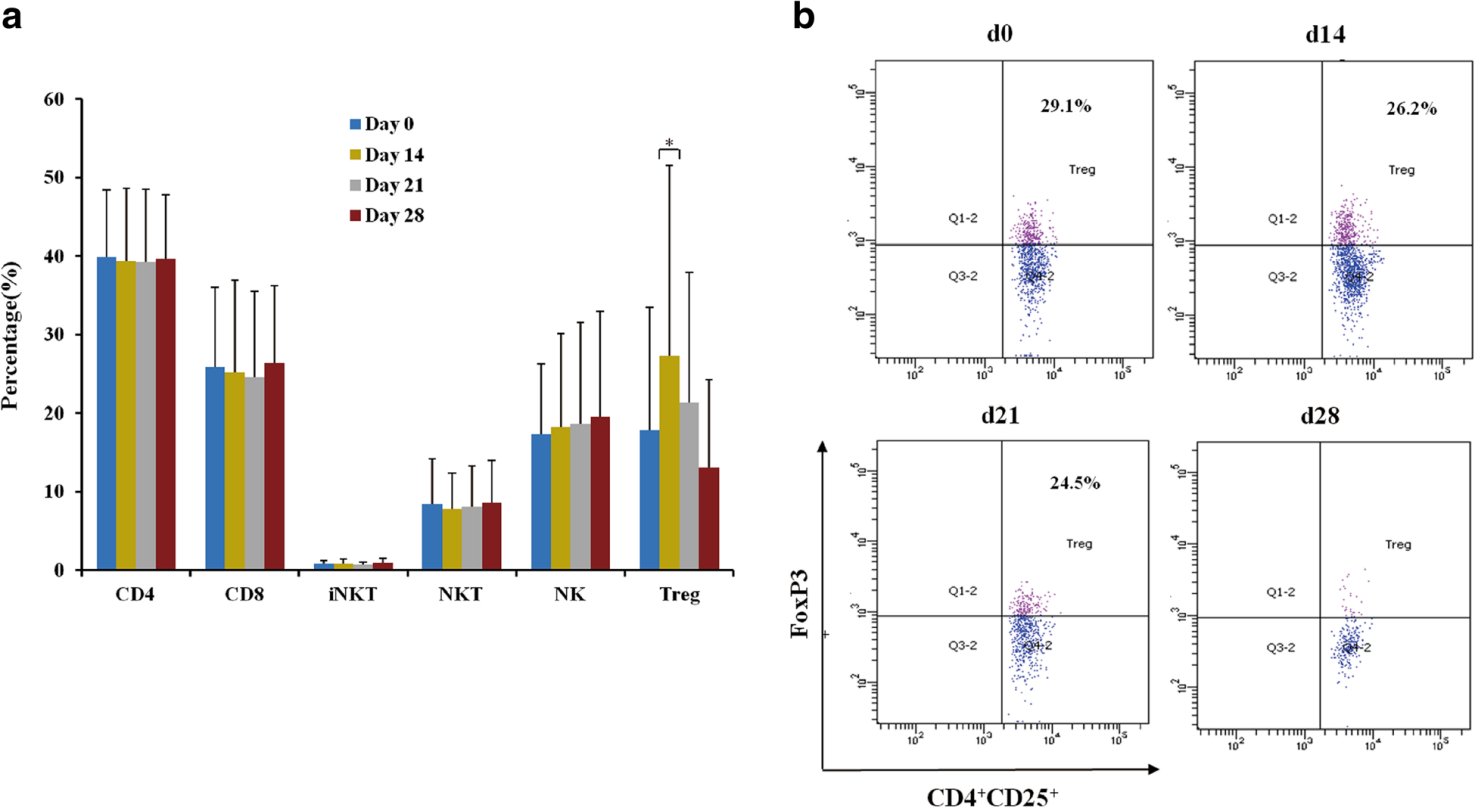
Lymphocyte subgroups with pre-vaccination (day 0) and post-vaccination (day 14, 21, and 28) as by flow cytometry. **a** The bar graph represent mean ± standard deviation (n = 15). The percentages of CD3^+^CD4^+^T cells,CD3^+^CD8^+^ T cell, CD3^+^CD56^+^ natural killer T (NKT) cell, CD3^+^Vα24^+^ iNKT cell, and CD3^−^CD16^+^CD56^+^ NK cell populations were not significantly increased (*p* > 0.05). The percentage of CD3^+^CD4^+^CD25^+^Foxp3^+^ T regulatory cells (Tregs) population was significantly decreased starting from day 14 (*, *p* < 0.05). **b** Representative dot plot (gated on CD3^+^CD4^+^CD25^+^) of CD3^+^CD4^+^CD25^+^Foxp3^+^ Tregs of post-and pre-vaccination

The lymphocyte subgroups and the production of cytokines of all 15 patients were analyzed for prior to and post the vaccination at day 14, 21, and 28. The T cell subgroups included CD3^+^CD4^+^, CD3^+^CD8^+^, CD3^+^CD56^+^, CD3^+^Vα24^+^, and CD3^+^CD4^+^CD25^+^Foxp3^+^, as well as CD3^−^CD16^+^CD56^+^ NK cells. The percentages of CD3^+^CD8^+^ T cells, CD3^+^CD56^+^ natural killer T (NKT) cells, CD3^+^Vα24^+^ iNKT cells, and CD3^−^CD16^+^CD56^+^ NK cells did not increased significantly(*p* > 0.05). No apparent change in the percentage of CD3^+^CD4^+^ (*p* > 0.05) was observed. However, the percentage of CD3^+^CD4^+^CD25^+^Foxp3^+^ T regulatory (Treg) cells was significantly decreased starting from day 14 (*p* < 0.05, Fig. 4).

**Fig. 5:**
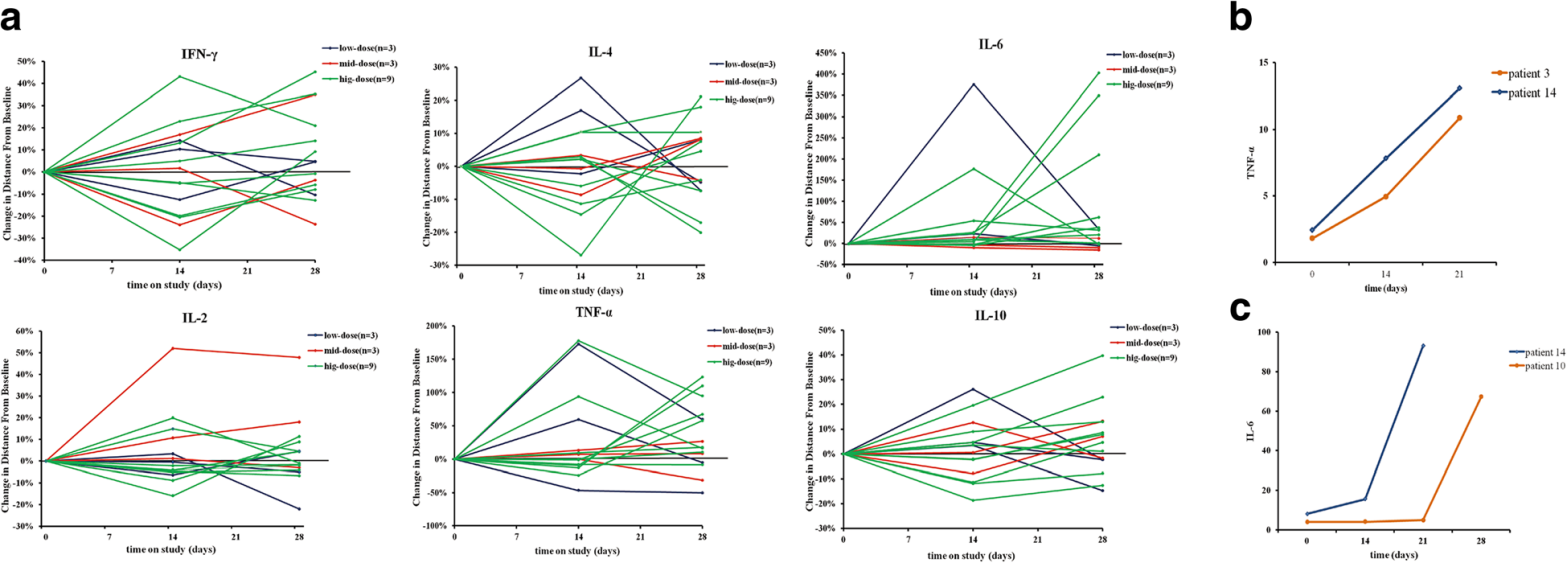
Levels of cytokines IL-2, IL-4, IL-6, IL-10, IFN-γ, and TNF-α. Percentage changes in individual cytokine levels are represented in line graphs (**a**). Comparisons of the leves of individual patients for TNF-α (**b**) and IL-6 (**c**) levels are indicated with respect to time

The cytokines IL-2, IL-4, IL-6, IL-10, IFN-γ, and TNF-α were observed in all the patients. The levels of IL-2, IL-4, IL-10, and IFN-γ were not significantly increased compared to pre-vaccination (*p* > 0.05, Fig. 5a). However, the TNF-α levels were significantly increased in 2 of 15 patients from 1.81 to 10.86 pg/mL and from 2.43 to 13.07 pg/mL, respectively (Fig. 5b). The IL-6 levels were also significantly increased in two patients from 4.03 to 67.23 and from 4.14 to 87.96, respectively (Fig. 5c).

### Clinical response

**Fig. 6:**
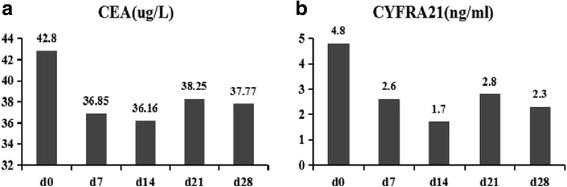
Tumor marker expression pre-vaccination at day 0 and post-vaccination at day 14, 21and 28. **a** The CEA levels under post-vaccination were decreased compared with pre-vaccination in patient 14. **b** The levels of CYFRA21 had decreased to normal at post-vaccination compared with pre-vaccination in patient 15

**Fig. 7:**
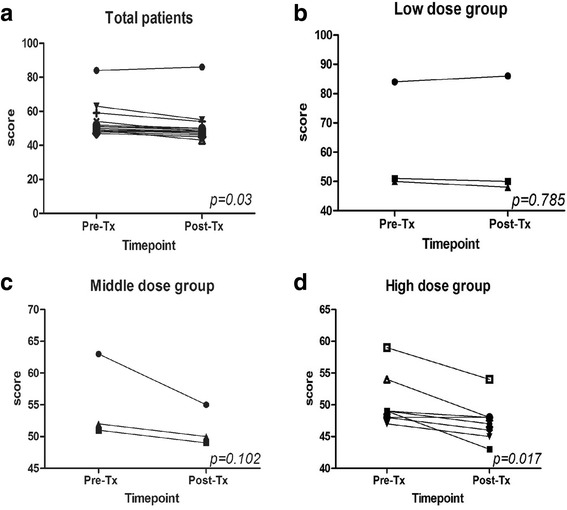
Score of patients’ quality of life. **a** All enrolled patients (*p* = 0.003). **b** Low dose group (*p* > 0.05). **c** Middle dose group (*p* > 0.05). **d** High dose group (*p* < 0.05). Each line represents one patient. Pre-Tx, pre-vaccination; Post-Tx,post-vaccination

In this phase I clinical trail, 15 patients with resected stage I to IIIA NSCLC were enrolled, all patients had no visible tumor lesions pre- and 1 month after vaccination injection. Mealwhlie, the tumor markers CEA, SCC, CYFRA21, and CA125 were analysed in all the patients.13 patients had normal tumor markers at baseline, and only 2 patients had abnormal tumor markers. The carcino-embryonic antigen (CEA) levels were decreased in patient No.14 patient (Fig. 6a), and the CYFRA21 levels were decreased to normal levels in No.15 patient after the vaccination (Fig. 6b). The other tumor markers remained almost at normal levels. Meanwhile, the patients’ quality of life improved after the vaccination as the score of the quality of life was significantly decreased, compared to pre-vaccination (*p* < 0.05). Furthermore, patients’ quality of life was significantly improved in the high-dose group, compared with low-dose and middle-dose groups after the treatment (*p* < 0.05, Fig. 7).

More importantly, in the long-term follow-up until 2017, 1 patient had no recurrence, 1 patients had a progressive disease (PD), and 1 patient was died on May 1, 2015 in the low dose group. In the middle dose group, all 3 patients had no recurrence. In the high dose group, 1 patient was died on 21 April, 2015, 1 patient had a PD, and the other 7 patients had no recurrence.

## Discussion

NSCLC accounts for 85% of lung cancer. Surgery following platinum-based chemotherapy and radiation is still the primary treatment for resectable stage I to IIIA NSCLC. However, the prognosis is poor, and 5-year survival rate of all stages is only 16%. Therefore, vaccination is the first and essential choice for NSCLC prevention. In this study, we developed a novel modified-DC vaccine that was pulsed with survivin and MUC1, silenced with SCOS1, and immune-stimulated with flagellin. We performed a phase I clinical trial of the vaccine in patients with resected NSCLC. The vaccine had a modest antitumor activity without dose-limiting toxicity. Notably, the positive cytokines were increased, and the negative lymphocytes were decreased as compared to baseline. Thus, vaccination with the modified-DC vaccine modulated the tumor microenvironment to elicit an immune response against the tumor. We found the CEA levels were decreased in No.14 patient, and the CYFRA21 returned to normal levels in No.15 patient. The quality of life of patients immunized with the maximum dose of vaccine was significantly improved after the vaccination. The novel DC vaccine enhanced DC differentiation and antigen presentation feature, and strongly activated antigen-specific T cell immune response by survivin and MUC1 pulsing and SOCS1 silencing and stimulation of TLR signaling.

The vaccination-related AEs were reported to be under lower grade category within the 28-day period. Therefore, this novel modified-DC vaccine did not exhibit unanticipated or serious AEs. Of note, the incidence of CRP increased, and the occurance of pyrexia and myalgia in the highest dose group were high. The patient numbers 10 and 14 experienced myalgia and pyrexia accompanied by increased CRP and had the highest temperature 38.9 °C and 38.8 °C, respectively. In fact, CRP, the major acute-phase reactant in humans, increases rapidly in response to inflammatory stimuli to recognize pathogens and damage cells [51]. Thus, better anti-tumor effects might be seen in patients who experienced major adverse events.

The two patients 10 and 14 who experienced myalgia and pyrexia accompanied by increased CRP also showed elevated IL-6 levels, with the highest levels 67.23 pg/mL and 87.96 pg/mL, respectively. This is in line with the findings in another study in which IL-6, as an important mediator of fever, is responsible for stimulating acute phase protein CRP synthesis [52]. On the one hand, IL-6 changes the temperature set point to increase body temperature [53] and stimulates energy mobilization in muscle and fatty tissues to increase body temperature. IL-6 is also a myokine that is produced from muscle and elevated in response to muscle contraction [54]. Meanwhile, IL-6 is secreted by T cells and macrophages in response to TLRs, leading to inflammatory cytokine production [55]. In addition, IL-6 also stimulates T lymphocyte proliferation, influences growth, differentiation, and migration of tumor cells, and stimulates angiogenesis [56]. Therefore, the elevated IL-6 levels secreted by T cells in patients 10 and 14 might indicate successful targeting of tumor cells. In fact, the increases in the inflammatory cytokines, and IFN-γ and IL-6 coincide with the onset of the tumor lysis syndrome after modified T cell infusion [57].

In this study, patients 10 and 3 after the vaccination had significant increases in IL-6 and TNF-α, respectively. Patient 14 featured increases in both IL-6 and TNF-α. The levels of IL-6 were 21 times higher than baseline levels, and the highest level of IL-6 showed up at 21 to 28 days after the first modified-DC vaccine infusion. The temporal rise in cytokine levels paralleled the clinical symptoms as mentioned above. The serum levels of IL-6 were increased after the vaccination, indicating the activation of T cells and induction of immune response by the host. This is consistent with the previous findings that SOCS1-silenced DCs could produce cytokines, including IFN-γ, IL-12, TNF-α, and IL-6 [36]. The cytokines secreted by immune cells play an important role in immunotherapy. Cytokine changes are used to represent the strength of immune response [58]. Type-1 T helper (Th1) cells mainly produce IL-2, IFN-γ, and TNF-α that drive cytotoxic T cell (CTL) response and induce high levels of anti-inflammatory response [59]. In contrast, type-2 T helper (Th2) cells produce IL-4, IL-6, and IL-10 that are responsible for humoral immunity to stimulate B cell proliferation and produce immunoglobulin [59]. The imbalance of Th1 and Th2 is related to tumor immune escape and the pathogenesis of various diseases [60]. TNF-α, another Th1 cytokine produced by activated T cells, is able to induce tumor cell necrosis and enhance the activity of NK and T cells [61].

Furthermore, the analysis of lymphocyte subsets is also a useful way to evaluate immune response [62]. The modified-DC vaccine was found to inhibit production of the Treg cell proliferation, which is an immune suppressing lymphocyte. There were no obvious changes in CD3^+^CD4^+^CD8^−^, CD3^+^CD4^−^CD8^+^, CD3^+^Vα24^+^, CD3^+^CD56^+^, orCD3^−^CD16^+^CD56^+^ cell population between the patients before and after vaccination, but CD3^+^CD4^+^CD25^+^Foxp3^+^ Treg cell number was significantly decreased after a transient increase at day 14 (*p* < 0.05). Treg cells play critical role in maintaining immune tolerance through suppressing the effector T cell responses as well as the activity of DC [63]. Increased number of Treg cells in multiple tumors is correlated with a poorer prognosis [64–66]. TLR5 agonists inhibit the function or number of Treg cells to suppress tumor growth [67].

While efficacy was not the primary end point of this trial, we found evidence of mild anti-tumor activity. In this phase I clinical trial, all the patients enrolled were not found visible tumor lesions in the baseline, and 1 month after treatment all patients did not get tumor recurrence. Meanwhile, we analysed the tumor markers such as CEA, SCC, CYFRA21, and CA125. 1 patient experienced a decrease in tumor marker CEA and the other had normal levels of tumor marker CYFRA21. It is interesting that patient 14 experienced a significant increase in CRP, IL-6, and TNF-α level, but a significant decrease in tumor marker CEA levels. It is possible that there was a combination of cytokine and lymphocyte induced clinical response. This may be the limitation of the DC vaccine. The small number of patients in this study is another potential limitation of this study.

Moreover, this modified DC vaccine maybe a potential treatment for patients with resected NSCLC to prevent recurrence. In all the patients, only 2 patients died about 2 years after injecting the vaccine, and 2 patients had a PD, 11 patients were still with recurrence-free survival (RFS). Also, there is another limitation of the current study which is that there is no potential way of knowing the effect of the vaccine on reducing tumor volume. In this clinical trial where no objective response rate (ORR) of the tumour occurred, the benefits could have been falsely attributed to the DC vaccine therapy.

## Conclusion

In conclusion, a novel survivin and MUC1 pulsed DC vaccine with silenced SOCS1 and stimulated TLR immune was developed in this study and exhibited convincing phase I trial outcomes in preventing NSCLC. Further more, the vaccine maybe a very efficacious treatment for patients with resected NSCLC to prevent recurrence. Our findings provide a potential DC vaccine for NSCLC, which is worthy of a future phase II/III clinical trial.

## Abbreviations

AEs: Adverse events
AJCC: American Joint Commission on Cancer
ANOVA: Analysis of variance
APC: Allophycocyanin
CEA: Carcino-embryonic antigen
CTCAE 4: Common Terminology Criteria for Adverse Events version 4
CTL: Cytotoxic T-lymphocyte
DC: Dendritic cell
ECOG PS: Eastern Cooperative Oncology Group Performance Status
FITC: Fluorescein isothiocyanate
IAP: Inhibitor of apoptosis protein
IFN-γ: Interferon gamma
IL-4: Interleukin 4
NKT: Natural killer T
NSCLC: Non-small cell lung cancer
PAP: Prostatic acid phosphatase
PBMCs: Peripheral blood mononuclear cells
PE: Phycoerythrin
SOSC1: Suppressor of cytokine signaling 1
TLR: Toll-like receptor
TNF-α: Necrosis factor alpha
TNM: Tumor Node Metastasis
Treg: T regulatory

## References

[1] Jemal A, Bray F, Center MM, Ferlay J, Ward E, Forman D (2011). Global cancer statistics. CA Cancer J Clin.

[2] Siegel R, Ma J, Zou Z, Jemal A (2014). Cancer statistics, 2014. CA Cancer J Clin.

[3] Garcia M, Jemal A, Ward E, Center M, Hao Y, Siegel R, Thun M (2007). Global Cancer Facts & Figures 2007. Atlanta.

[4] Rami-Porta R, Crowley JJ, Goldstraw P (2009). Review the revised TNM staging system for lung cancer. Ann Thorac Cardiovasc Surg.

[5] Scagliotti GV, Parikh P, Von Pawel J, Biesma B, Vansteenkiste J, Manegold C, Serwatowski P, Gatzemeier U, Digumarti R, Zukin M, Phase III (2008). Study comparing cisplatin plus gemcitabine with cisplatin plus pemetrexed in chemotherapy-naive patients with advanced-stage non–small-cell lung cancer. J Clin Oncol.

[6] Schiller JH, Harrington D, Belani CP, Langer C, Sandler A, Krook J, Zhu J, Johnson DH (2002). Comparison of four chemotherapy regimens for advanced non–small-cell lung cancer. N Engl J Med.

[7] Mok TS, Y-L W, Thongprasert S, Yang C-H, Chu D-T, Saijo N, Sunpaweravong P, Han B, Margono B, Ichinose Y (2009). Gefitinib or carboplatin–paclitaxel in pulmonary adenocarcinoma. N Engl J Med.

[8] Shepherd FA, Rodrigues Pereira J, Ciuleanu T, Tan EH, Hirsh V, Thongprasert S, Campos D, Maoleekoonpiroj S, Smylie M, Martins R (2005). Erlotinib in previously treated non–small-cell lung cancer. N Engl J Med.

[9] Shaw AT, Yeap BY, Solomon BJ, Riely GJ, Gainor J, Engelman JA, Shapiro GI, Costa DB, S-HI O, Butaney M (2011). Effect of crizotinib on overall survival in patients with advanced non-small-cell lung cancer harbouring ALK gene rearrangement: a retrospective analysis. Lancet Oncol.

[10] Sandler A, Gray R, Perry MC, Brahmer J, Schiller JH, Dowlati A, Lilenbaum R, Johnson DH (2006). Paclitaxel–carboplatin alone or with bevacizumab for non–small-cell lung cancer. N Engl J Med.

[11] Prestwich R, Vile R, Melcher A. Cancer immunotherapy. N Engl J Med 2008; 359: 1072; author reply 3. 18777615

[12] Kantoff PW, Higano CS, Shore ND, Berger ER, Small EJ, Penson DF, Redfern CH, Ferrari AC, Dreicer R, Sims RB, Xu Y, Frohlich MW, Schellhammer PF. Sipuleucel-T Immunotherapy for castration-resistant prostate cancer. N Engl J Med 2010; 363: 411–422. 10.1056/NEJMoa1001294 20818862

[13] Chang CN, Huang YC, Yang DM, Kikuta K, Wei KJ, Kubota T, Yang WKA (2011). Phase I/II clinical trial investigating the adverse and therapeutic effects of a postoperative autologous dendritic cell tumor vaccine in patients with malignant glioma. J Clin Neurosci.

[14] Oshita C, Takikawa M, Kume A, Miyata H, Ashizawa T, Iizuka A, Kiyohara Y, Yoshikawa S, Tanosaki R, Yamazaki N, Yamamoto A, Takesako K, Yamaguchi K, Akiyama Y (2012). Dendritic cell-based vaccination in metastatic melanoma patients: phase II clinical trial. Oncol Rep.

[15] El Ansary M, Mogawer S, Elhamid SA, Alwakil S, Aboelkasem F, Sabaawy HE, Abdelhalim O (2013). Immunotherapy by autologous dendritic cell vaccine in patients with advanced HCC. J Cancer Res Clin Oncol.

[16] Fujiwara S, Wada H, Miyata H, Kawada J, Kawabata R, Nishikawa H, Gnjatic S, Sedrak C, Sato E, Nakamura Y, Sakakibara M, Kanto T, Shimosegawa E, Hatazawa J, Takahashi T, Kurokawa Y, Yamasaki M, Nakajima K, Takiguchi S, Nakayama E, Mori M, Doki Y (2012). Clinical trial of the intratumoral administration of labeled DC combined with systemic chemotherapy for esophageal cancer. J Immunother.

[17] Narita M, Kanda T, Abe T, Uchiyama T, Iwafuchi M, Zheng Z, Liu A, Kaifu T, Kosugi S, Minagawa M, Itoh K, Takahashi M (2015). Immune responses in patients with esophageal cancer treated with SART1 peptide-pulsed dendritic cell vaccine. Int J Oncol.

[18] Ambrosini G, Adida C, Altieri DCA (1997). Novel anti-apoptosis gene, survivin, expressed in cancer and lymphoma. Nat Med.

[19] Mita AC, Mita MM, Nawrocki ST, Giles FJ (2008). Survivin: key regulator of mitosis and apoptosis and novel target for cancer therapeutics. Clin Cancer Res.

[20] Fraunholz I, Rodel C, Distel L, Rave-Frank M, Kohler D, Falk S, Rodel F (2012). High survivin expression as a risk factor in patients with anal carcinoma treated with concurrent chemoradiotherapy. Radiat Oncol.

[21] Duan L, Hu X, Jin Y, Liu R, You Q (2016). Survivin protein expression is involved in the progression of non-small cell lung cancer in Asians: a meta-analysis. BMC Cancer.

[22] Liu TC, Hsieh MJ, WJ W, Chou YE, Chiang WL, Yang SF, SC S, Tsao TC (2016). Association between survivin genetic polymorphisms and epidermal growth factor receptor mutation in non-small-cell lung cancer. Int J Med Sci.

[23] Cho HJ, Kim HR, Park YS, Kim YH, Kim DK, Park SI (2015). Prognostic value of survivin expression in stage III non-small cell lung cancer patients treated with platinum-based therapy. J Surg Oncol.

[24] Giaccone G, Zatloukal P, Roubec J, Floor K, Musil J, Kuta M, van Klaveren RJ, Chaudhary S, Gunther A, Shamsili S (2009). Multicenter phase II trial of YM155, a small-molecule suppressor of survivin, in patients with advanced, refractory, non-small-cell lung cancer. J Clin Oncol.

[25] Kaira K, Nakagawa K, Ohde Y, Okumura T, Takahashi T, Murakami H, Endo M, Kondo H, Nakajima T, Yamamoto N. Depolarized MUC1 expression is closely associated with hypoxic markers and poor outcome in resected non-small cell lung cancer. Int J Surg Pathol 2012; 20: 223–232. 10.1177/1066896911429296 22108499

[26] Zhu WF, Li J, LC Y, Wu Y, Tang XP, YM H, Chen YC (2014). Prognostic value of EpCAM/MUC1 mRNA-positive cells in non-small cell lung cancer patients. Tumour Biol.

[27] Deng J, Wang L, Chen H, Li L, Ma Y, Ni J, Li Y (2013). The role of tumour-associated MUC1 in epithelial ovarian cancer metastasis and progression. Cancer Metastasis Rev.

[28] Woenckhaus M, Merk J, Stoehr R, Schaeper F, Gaumann A, Wiebe K, Hartmann A, Hofstaedter F, Dietmaier W (2008). Prognostic value of FHIT, CTNNB1, and MUC1 expression in non-small cell lung cancer. Hum Pathol.

[29] Lepisto AJ, Moser AJ, Zeh H, Lee K, Bartlett D, McKolanis JR, Geller BA, Schmotzer A, Potter DP, Whiteside T, Finn OJ, Ramanathan RKA (2008). Phase I/II study of a MUC1 peptide pulsed autologous dendritic cell vaccine as adjuvant therapy in patients with resected pancreatic and biliary tumors. J Cancer Ther.

[30] Kondo H, Hazama S, Kawaoka T, Yoshino S, Yoshida S, Tokuno K, Takashima M, Ueno T, Hinoda Y, Oka M (2008). Adoptive immunotherapy for pancreatic cancer using MUC1 peptide-pulsed dendritic cells and activated T lymphocytes. Anticancer Res.

[31] Chen J, Guo XZ, Li HY, Liu X, Ren LN, Wang D, Zhao JJ (2013). Generation of CTL responses against pancreatic cancer in vitro using dendritic cells co-transfected with MUC4 and survivin RNA. Vaccine.

[32] Aldrich M, Sanders D, Lapteva N, Huang XF, Chen SY (2008). SOCS1 downregulation in dendritic cells promotes memory T-cell responses. Vaccine.

[33] Wang Y, Wang S, Ding Y, Ye Y, Xu Y, He H, Li Q, Mi Y, Guo C, Lin Z, Liu T, Zhang Y, Chen Y, Yan JA (2013). Suppressor of cytokine signaling 1 antagonist enhances antigen-presenting capacity and tumor cell antigen-specific cytotoxic T lymphocyte responses by human monocyte-derived dendritic cells. Clin Vaccine Immunol.

[34] Hong B, Ren W, Song XT, Evel-Kabler K, Chen SY, Huang XF (2009). Human suppressor of cytokine signaling 1 controls immunostimulatory activity of monocyte-derived dendritic cells. Cancer Res.

[35] Evel-Kabler K, Song XT, Aldrich M, Huang XF, Chen SY (2006). SOCS1 restricts dendritic cells' ability to break self tolerance and induce antitumor immunity by regulating IL-12 production and signaling. J Clin Invest.

[36] Zhu Y, Zheng Y, Mei L, Liu M, Li S, Xiao H, Zhu H, Wu S, Chen H, Huang L (2013). Enhanced immunotherapeutic effect of modified HPV16 E7-pulsed dendritic cell vaccine by an adeno-shRNA-SOCS1 virus. Int J Oncol.

[37] Song S, Wang Y, Wang J, Lian W, Liu S, Zhang Z, Liu F, Wei L (2012). Tumour-derived IL-10 within tumour microenvironment represses the antitumour immunity of Socs1-silenced and sustained antigen expressing DCs. Eur J Cancer.

[38] Subramanya S, Armant M, Salkowitz JR, Nyakeriga AM, Haridas V, Hasan M, Bansal A, Goepfert PA, Wynn KK, Ladell K, Price DA, Manjunath N, Kan-Mitchell J, Shankar P (2010). Enhanced induction of HIV-specific cytotoxic T lymphocytes by dendritic cell-targeted delivery of SOCS-1 siRNA. Mol Ther.

[39] Su L, Sun Y, Ma F, Lu P, Huang H, Zhou J (2009). Progesterone inhibits toll-like receptor 4-mediated innate immune response in macrophages by suppressing NF-kappaB activation and enhancing SOCS1 expression. Immunol Lett.

[40] Zhang J, Li H, Yu JP, Wang SE, Ren XB (2012). Role of SOCS1 in tumor progression and therapeutic application. Int J Cancer.

[41] Hong B, Lee SH, Song XT, Jones L, Machida K, Huang XF, Chen SYA (2012). Super TLR agonist to improve efficacy of dendritic cell vaccine in induction of anti-HCV immunity. PLoS One.

[42] Oh DR, Kang HW, Kim JR, Kim S, Park IK, Rhee JH, Oh WK, Kim YR (2014). PMA induces vaccine adjuvant activity by the modulation of TLR signaling pathway. Mediators Inflamm.

[43] Polycarpou A, Holland MJ, Karageorgiou I, Eddaoudi A, Walker SL, Willcocks S, Lockwood DN (2016). Mycobacterium leprae activates toll-like Receptor-4 signaling and expression on macrophages depending on previous bacillus Calmette-Guerin vaccination. Front Cell Infect Microbiol.

[44] Leigh ND, Bian G, Ding X, Liu H, Aygun-Sunar S, Burdelya LG, Gudkov AV, Cao X (2014). A flagellin-derived toll-like receptor 5 agonist stimulates cytotoxic lymphocyte-mediated tumor immunity. PLoS One.

[45] Yanai S, Tokuhara D, Tachibana D, Saito M, Sakashita Y, Shintaku H, Koyama M (2016). Diabetic pregnancy activates the innate immune response through TLR5 or TLR1/2 on neonatal monocyte. J Reprod Immunol.

[46] Liu B, Chen S, Guan Y, Chen L, Type III (2015). Interferon induces distinct SOCS1 expression pattern that contributes to delayed but prolonged activation of Jak/STAT signaling pathway: implications for treatment non-response in HCV patients. PLoS One.

[47] Ren JP, Ying RS, Cheng YQ, Wang L, El Gazzar M, Li GY, Ning SB, Moorman JP, Yao ZQ (2016). HCV-induced miR146a controls SOCS1/STAT3 and cytokine expression in monocytes to promote regulatory T-cell development. J Viral Hepat.

[48] Edge SB, Compton CC (2010). The American joint committee on cancer: the 7th edition of the AJCC cancer staging manual and the future of TNM. Ann Surg Oncol.

[49] Storer BE (2001). An evaluation of phase I clinical trial designs in the continuous dose-response setting. Stat Med.

[50] Isla Larrain M, Demichelis S, Crespo M, Lacunza E, Barbera A, Creton A, Terrier F, Segal-Eiras A, Croce MV (2009). Breast cancer humoral immune response: involvement of Lewis y through the detection of circulating immune complexes and association with mucin 1 (MUC1). J Exp Clin Cancer Res.

[51] Rattazzi M, Puato M, Faggin E, Bertipaglia B, Zambon A, Pauletto P (2003). C-Reactive protein and interleukin-6 in vascular disease: culprits or passive bystanders?. Int J Hypertens.

[52] Banks WA, Kastin AJ, Gutierrez EG (1994). Penetration of interleukin-6 across the murine blood-brain barrier. Neurosci Lett.

[53] Febbraio MA, Pedersen BK (2005). Contraction-induced myokine production and release: is skeletal muscle an endocrine organ?. Exerc Sport Sci Rev.

[54] Chmielewski M, Abken HCART (2012). Cells transform to trucks: chimeric antigen receptor-redirected T cells engineered to deliver inducible IL-12 modulate the tumour stroma to combat cancer. Cancer Immunol Immunother.

[55] Nish SA, Schenten D, Wunderlich FT, Pope SD, Gao Y, Hoshi N, Yu S, Yan X, Lee HK, Pasman L, Brodsky I, Yordy B, Zhao H, Bruning J, Medzhitov RT (2014). Cell-intrinsic role of IL-6 signaling in primary and memory responses. elife.

[56] Maude SL, Frey N, Shaw PA, Aplenc R, Barrett DM, Bunin NJ, Chew A, Gonzalez VE, Zheng Z, Lacey SF, Mahnke YD, Melenhorst JJ, Rheingold SR, Shen A, Teachey DT, Levine BL, June CH, Porter DL, Grupp SA.. Chimeric Antigen Receptor T Cells for Sustained Remissions in Leukemia. N Engl J Med. 2014;371:1507–17. 10.1056/NEJMoa1407222 PMC4267531 25317870

[57] Keilholz U, Weber J, Finke JH, Gabrilovich DI, Kast WM, Disis ML, Kirkwood JM, Scheibenbogen C, Schlom J, Maino VC, Lyerly HK, Lee PP, Storkus W, Marincola F, Worobec A, Atkins MB (2002). Immunologic monitoring of cancer vaccine therapy: results of a workshop sponsored by the Society for Biological Therapy. J Immunother.

[58] Manocha GD, Floden AM, Puig KL, Nagamoto-Combs K, Scherzer CR, Combs CK (2017). Defining the contribution of neuroinflammation to Parkinson's disease in humanized immune system mice. Mol Neurodegener.

[59] Wang GZ, Cheng X, Zhou B, Wen ZS, Huang YC, Chen HB, Li GF, Huang ZL, Zhou YC, Feng L, Wei MM, LW Q, Cao Y, Zhou GB (2015). The chemokine CXCL13 in lung cancers associated with environmental polycyclic aromatic hydrocarbons pollution. elife.

[60] Xu Y, Gao J, Su Z, Dai X, Li Y, Liu Y, Chen J, Tong J, Zhang Y, Wu C, Zheng D, Wang S, Xu H (2012). Downregulation of Hlx closely related to the decreased expressions of T-bet and Runx3 in patients with gastric cancer may be associated with a pathological event leading to the imbalance of Th1/Th2. Clin Dev Immunol.

[61] Whiteside TL, Regulatory T (2014). Cell subsets in human cancer: are they regulating for or against tumor progression?. Cancer Immunol Immunother.

[62] Lin X, Chen M, Liu Y, Guo Z, He X, Brand D, Zheng SG (2013). Advances in distinguishing natural from induced Foxp3(+) regulatory T cells. Int J Clin Exp Pathol.

[63] Zhang B, Chikuma S, Hori S, Fagarasan S, Honjo T (2016). Nonoverlapping roles of PD-1 and FoxP3 in maintaining immune tolerance in a novel autoimmune pancreatitis mouse model. Proc Natl Acad Sci U S A.

[64] Idris SZ, Hassan N, Lee LJ, Md Noor S, Osman R, Abdul-Jalil M, Nordin AJ, Abdullah M (2016). Increased regulatory T cells in acute lymphoblastic leukaemia patients. Hematology.

[65] Wang Y, Sun J, Zheng R, Shao Q, Gao W, Song B, Chen X, Regulatory QX (2016). T cells are an important prognostic factor in breast cancer: a systematic review and meta-analysis. Neoplasma.

[66] Tang Y, Xu X, Guo S, Zhang C, Tang Y, Tian Y, Ni B, Lu B, Wang H (2014). An increased abundance of tumor-infiltrating regulatory T cells is correlated with the progression and prognosis of pancreatic ductal adenocarcinoma. PLoS One.

[67] Gong XD, Ma LM, Zhu L, Guo HM, Ren LS, Ren RR, Zhang HP, Wei F, Niu YY (2012). Prophylactic effect of TLR5 agonist flagellin on acute graft versus host disease after allogeneic hematopoietic stem cell transplantation and its mechanism. Zhongguo shi yan xue ye xue za zhi.

